# Pancreatic T cell protein-tyrosine phosphatase deficiency ameliorates cerulein-induced acute pancreatitis

**DOI:** 10.1186/1478-811X-12-13

**Published:** 2014-03-10

**Authors:** Ahmed Bettaieb, Yannan Xi, Ellen Hosein, Nicole Coggins, Santana Bachaalany, Florian Wiede, Salvador Perez, Stephen M Griffey, Juan Sastre, Tony Tiganis, Fawaz G Haj

**Affiliations:** 1 Department of Nutrition, University of California Davis, One Shields Ave, 3135 Meyer Hall, Davis, CA 95616, USA; 2 Department of Biochemistry and Molecular Biology, Monash University, Melbourne, Victoria, Australia; 3 Department of Physiology, University of Valencia, 46100 Burjasot, Valencia, Spain; 4 Comprehensive Pathology Laboratory, University of California Davis, Davis, CA 95616, USA; 5 Division of Endocrinology, Diabetes and Metabolism, Department of Internal Medicine, University of California Davis, Sacramento, CA 95817, USA; 6 Comprehensive Cancer Center, University of California Davis, Sacramento, CA 95817, USA

**Keywords:** Acute pancreatitis, TCPTP, Inflammation, STAT3, Knockout mice

## Abstract

**Background:**

Acute pancreatitis (AP) is a common clinical problem whose incidence has been progressively increasing in recent years. Onset of the disease is trigged by intra-acinar cell activation of digestive enzyme zymogens that induce autodigestion, release of pro-inflammatory cytokines and acinar cell injury. T-cell protein tyrosine phosphatase (TCPTP) is implicated in inflammatory signaling but its significance in AP remains unclear.

**Results:**

In this study we assessed the role of pancreatic TCPTP in cerulein-induced AP. TCPTP expression was increased at the protein and messenger RNA levels in the early phase of AP in mice and rats. To directly determine whether TCPTP may have a causal role in AP we generated mice with pancreatic TCPTP deletion (panc-TCPTP KO) by crossing TCPTP floxed mice with Pdx1-Cre transgenic mice. Amylase and lipase levels were lower in cerulein-treated panc-TCPTP KO mice compared with controls. In addition, pancreatic mRNA and serum concentrations of the inflammatory cytokines TNFα and IL-6 were lower in panc-TCPTP KO mice. At the molecular level, panc-TCPTP KO mice exhibited enhanced cerulein-induced STAT3 Tyr705 phosphorylation accompanied by a decreased cerulein-induced NF-κB inflammatory response, and decreased ER stress and cell death.

**Conclusion:**

These findings revealed a novel role for pancreatic TCPTP in the progression of cerulein-induced AP.

## Background

Acute pancreatitis (AP) is often the most common reason for hospitalization from gastrointestinal diseases in Western countries with an unpredictable clinical course [1,2]. The incidence of AP has been progressively increasing in recent years in parallel with its risk factors such as duct obstruction by gallstones, alcohol abuse and obesity [2,3]. Approximately 25% of patients with AP develop a severe disease course that leads to systemic inflammation and multiple organ dysfunction with mortality rates of up to 50% [2,4]. The onset of the disease is triggered by acinar events that involve premature intra-acinar activation of digestive enzymes such as trypsinogen that induces autodigestion, the release of pro-inflammatory cytokines and acinar cell injury [5-7]. AP remains without specific therapy and understanding the molecular mechanisms underlying its pathogenesis will aid in therapeutic intervention.

Several animal models of AP have been generated to investigate the pathogenesis and to explore potential therapeutic approaches; one of the most common is cerulein-induced pancreatitis [8]. Cerulein is an ortholog of the intestinal hormone cholecystokinin and at high concentrations causes pancreatic secretion of lipase and amylase, death of acinar cells, edema formation and the infiltration of inflammatory cells into the pancreas [9-11], all of which are also observed in human pancreatitis. The mechanism of cerulein action involves activation of NF-κB, the promotion of oxidative stress, and the release of pro-inflammatory cytokines [12,13]. In addition, cerulein treatment modulates pancreatic protein tyrosine kinase (PTK) and protein tyrosine phosphatase (PTP) activities [14,15].

The roles of PTPs in AP remain largely unexplored, but some studies have demonstrated altered PTPs expression and activity in murine models of AP. Indeed, cerulein-induced AP in rats is associated with increases in the expression of SHP1 and SHP2 and changes in the dynamics of SHP2 subcellular distribution during the early phase of AP progression [16]. In addition, expression of the endoplasmic reticulum (ER)-anchored protein phosphatase PTP1B is increased in the early phase of cerulein-induced AP [17]. Although these findings suggest a role for PTPs in AP, additional investigation into the contribution of PTPs to the pathogenesis of AP is warranted.

T-cell protein tyrosine phosphatase (TCPTP; encoded by *PTPN2*) is a ubiquitously-expressed PTP [18]. Two splice variants of TCPTP are expressed: a 48 kDa form which is anchored to the ER by a hydrophobic C-terminus, and a 45 kDa variant that lacks the hydrophobic C-terminus and has access to nuclear and cytosolic substrates [19-21]. Several substrates of TCPTP have been identified and include receptor PTKs (RTKs) [19,22,23], non-receptor PTKs such as c-Src [24] and Janus family kinases (JAKs) 1/3 [25], and substrates of PTKs such as signal transducer and activator of transcription (STAT) 1, 3, 5 and 6 [26-30]. Whole-body TCPTP deficiency in mice leads to hematopoietic defects and progressive systemic inflammatory disease [31,32]. More recently, tissue-specific TCPTP deletion helped define the functions of this phosphatase in T cells [33], muscle [34] and brain [30]. However, the function of TCPTP in the pancreas remains unresolved. TCPTP is expressed in the endocrine and exocrine pancreas in mice with stronger expression in islets than the surrounding exocrine tissue [35]. Genome-wide association screens identify *PTPN2* as a susceptibility gene in the pathogenesis of type 1 diabetes [36] whereas others report that TCPTP regulates cytokine-induced β-cell apoptosis [37,38]. In addition, TCPTP regulates ER stress in the glucose-responsive MIN6 β-cells and alterations in pancreatic TCPTP expression may serve as an adaptive response for the mitigation of chronic ER stress [35].

In the present study, we investigated the effects of pancreatic TCPTP deletion on cerulein-induced AP. Alterations in systemic inflammation were determined in cerulein-treated versus non-treated control and pancreas-TCPTP knockout mice, and the underlying molecular mechanism investigated.

## Results

### TCPTP expression is increased in the early phase of acute pancreatitis

**Figure 1:**
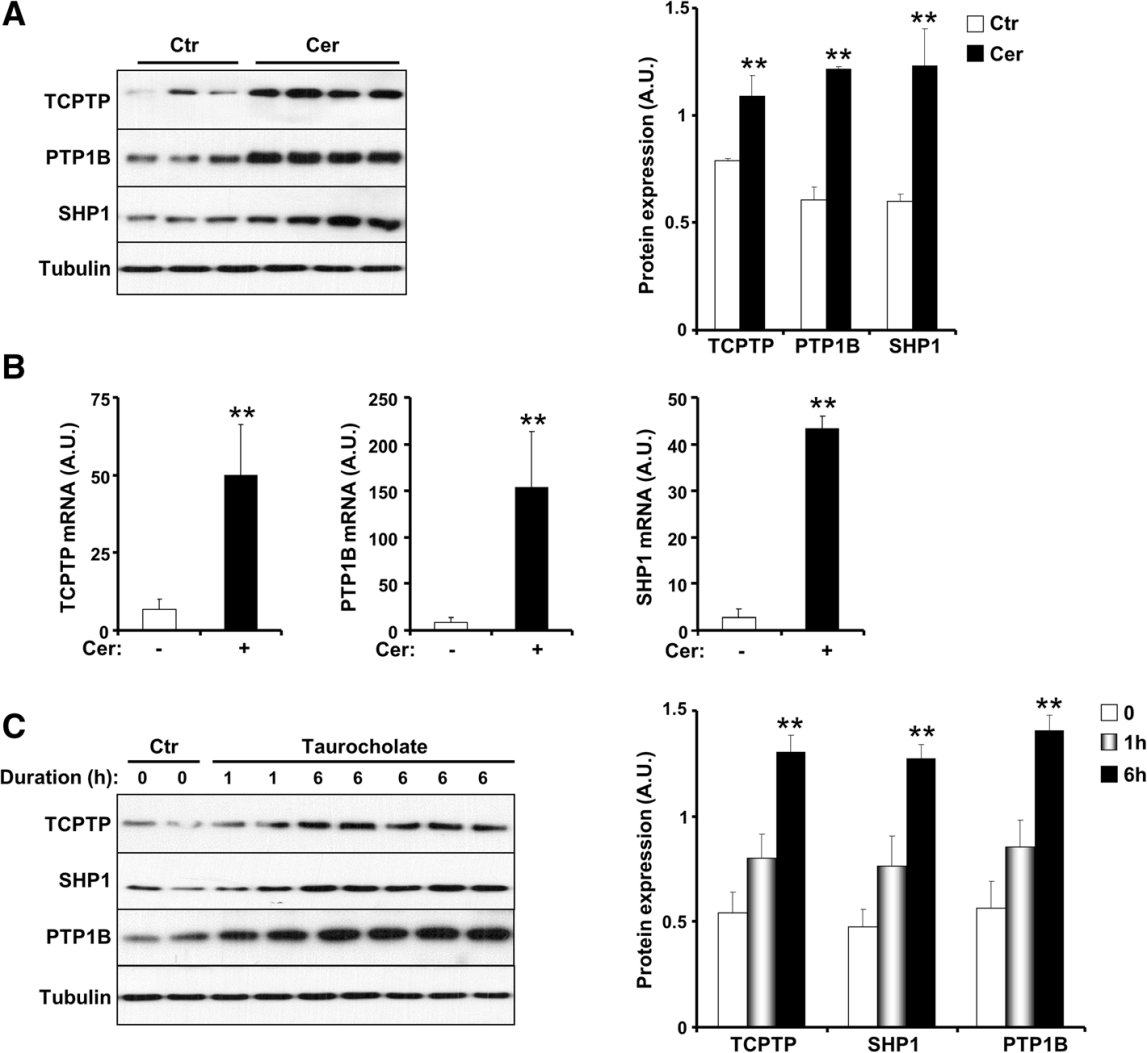
**Increased TCPTP expression in acute pancreatitis. A)** Total pancreas lysates of wild type mice that were administered saline (control; Ctr; n = 9) or cerulein (Cer; n = 12) immunoblotted for TCPTP, PTP1B, SHP1 and Tubulin. Bar graph represents expression of TCPTP, PTP1B and SHP1 (normalized to Tubulin) and presented as means ± SEM. **B)***TCPTP, PTP1B* and *SHP1* expression as assessed by quantitative real time PCR in the pancreata of wild type mice without (-) (n = 6) and with (+) (n = 8) cerulein administration. For A and B (**; P ≤ 0.01) indicates significant difference between saline- and cerulein-injected mice. **C)** Total pancreas lysates of rats that were administered saline or taurocholate, sacrificed after 1 and 6 h then immunoblotted for TCPTP, PTP1B, SHP1 and Tubulin. Bar graph represents expression of TCPTP, PTP1B and SHP1 (normalized to Tubulin) and presented as means ± SEM. (**; P ≤ 0.01) indicates significant difference between saline- and taurocholate-injected rats.

AP was induced by repetitive intraperitoneal injections of cerulein, an analog of the secretagogue cholecystokinin, to wild type mice and expression of TCPTP was determined. Immunoblots of pancreatic lysates demonstrated increased TCPTP expression upon cerulein administration (Figure 1A). Expression of the closely related PTP1B and the SH2 domain-containing phosphatase SHP1 was increased upon cerulein administration consistent with published reports [16,17]. In addition, mRNAs of the genes encoding TCPTP, PTP1B and SHP1, as determined by real time RT-PCR, were increased in the pancreas upon cerulein administration (Figure 1B). Similarly, pancreatic TCPTP, SHP1 and PTP1B protein expression was increased in a taurocholate-induced AP rat model [39,40] (Figure 1C). Together, these findings demonstrate that AP is associated with increases in TCPTP at the level of both mRNA and protein.

### Ablation of pancreatic TCPTP mitigates cerulein-induced pancreatitis

**Figure 2:**
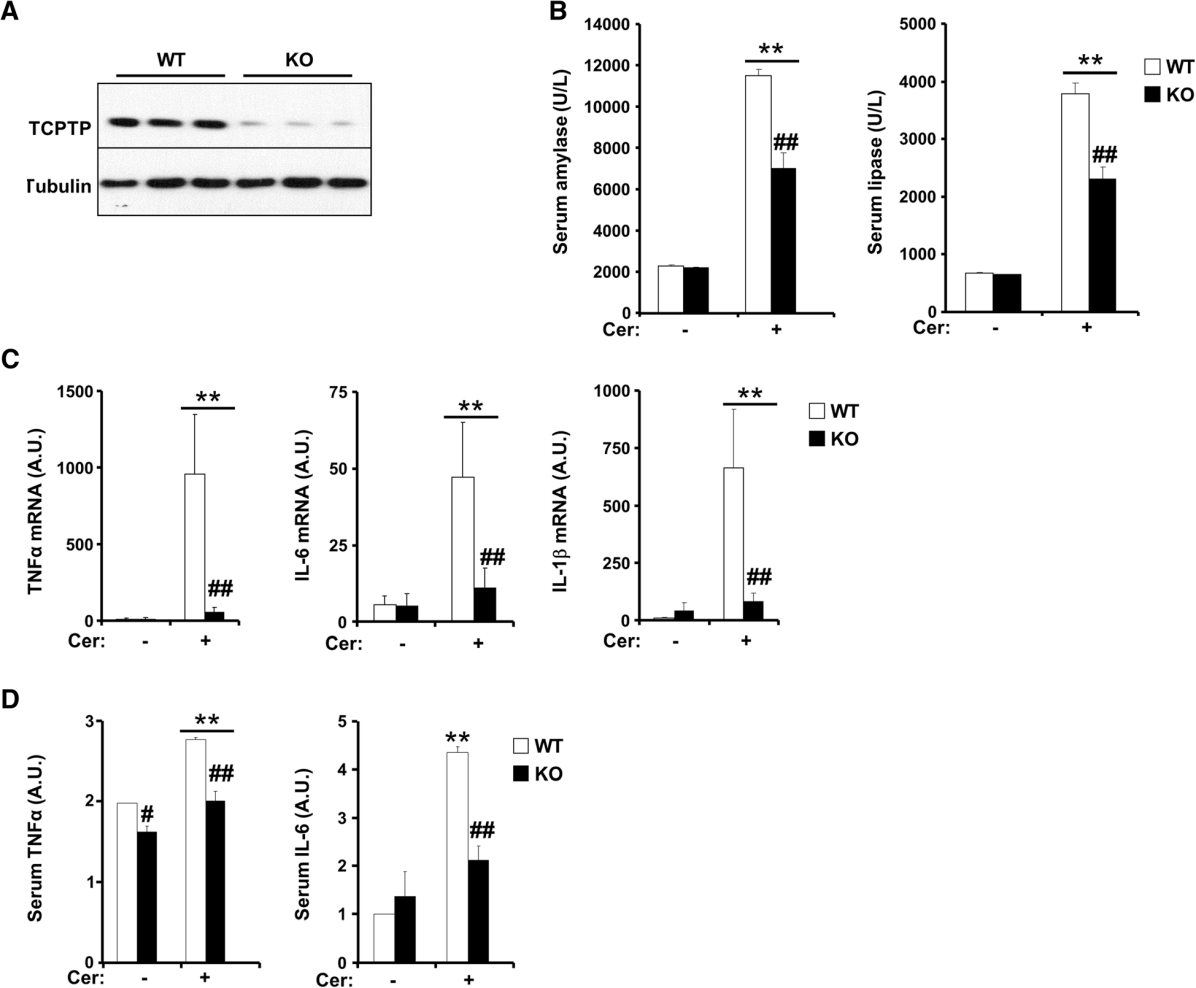
**Pancreatic TCPTP deficiency decreases cerulein-induced pancreatic injury. A)** Total pancreas lysates from wild type (WT) and panc-TCPTP KO (KO) mice were immunoblotted for TCPTP and Tubulin. **B)** Acute pancreatitis was induced by intraperitoneal injections of cerulein as detailed in Methods. Serum amylase and lipase were determined in control mice without (n = 9) and with (n = 12) cerulein and in panc-TCPTP KO mice without (n = 7) and with (n = 9) cerulein. **C)***TNFα*, *IL-6* and *IL-1β* expression (as assessed by quantitative real time PCR) in the pancreata of control mice without (n = 6) and with (n = 6) cerulein and panc-TCPTP KO mice without (n = 5) and with (n = 5) cerulein. **D)** Circulating levels of TNFα and IL-6 in serum of control mice without (n = 6) and with (n = 12) cerulein and panc-TCPTP KO mice without (n = 6) and with (n = 6) cerulein. (**; P ≤ 0.01) indicates significant difference between saline- and cerulein-injected mice, and (##; P ≤ 0.01) indicates significant difference between WT and KO mice.

The increased expression of TCPTP upon cerulein administration prompted us to investigate the role of this phosphatase in AP. To that end, we crossed TCPTP^fl/fl^ mice to those expressing Cre recombinase under the control of pancreatic and duodenal homeobox 1 (Pdx1) promoter to generate mice lacking TCPTP in the (endocrine and exocrine) pancreas [41]. Pancreatic TCPTP knockout mice (hereafter referred to as panc-TCPTP KO) survived to adulthood and did not display gross defects in pancreatic development. Immunoblot analysis of total pancreas lysates demonstrated significant reduction in TCPTP expression in panc-TCPTP KO mice compared with controls (Figure 2A). In addition, TCPTP expression was unchanged in other tissues such as hypothalamus, liver, muscle and adipose tissue (data not shown, and Xi *et al.*, submitted). Similar to wild type mice, panc-TCPTP KO mice exhibited increased expression of SHP1 and PTP1B upon cerulein administration (Additional file 1: Figure S1). Thus, this mouse model provides efficient TCPTP deletion in the pancreas enabling the determination of TCPTP contribution to pancreatitis.

To clarify the significance of TCPTP during AP, we determined the severity of cerulein-induced pancreatitis in control and panc-TCPTP KO mice. Mice were fasted overnight and cerulein (50 μg/kg body weight) administered over 12 h and analyses undertaken 2 h later. Histological analysis (Hematoxylin and Eosin staining) evaluating pathologic changes including edema, cell vacuolation and necrosis did not reveal any overt differences between cerulein-treated and untreated mice in this acute timeframe between treatment and euthanasia (data not shown). However, serum activities of amylase and lipase that are commonly used as markers for pancreatic disease, particularly AP were significantly different between control and panc-TCPTP KO mice with and without cerulein administration. Under basal conditions, serum amylase and lipase were comparable between control and panc-TCPTP KO mice (Figure 2B). Cerulein administration led to significant increase in amylase and lipase; however pancreatic TCPTP deficiency significantly reduced amylase and lipase after cerulein administration. Comparable findings were observed in two independent cohorts of mice. During AP the activation of NF-κB enhances the release of many pro-inflammatory cytokines such as TNFα, IL-1β and IL-6. TNFα, IL-1β are considered primary cytokines in AP since they initiate and propagate most of the consequences of the systemic inflammatory response [42,43], while IL-6 mediates the acute-phase response [44]. Accordingly, pancreatic mRNA levels of TNFα, IL-6 and IL-1β were increased in control mice after cerulein administration and this was significantly reduced in panc-TCPTP KO (Figure 2C). Similarly, serum levels of TNFα and IL-6 were increased in control mice after cerulein administration and this was significantly reduced in panc-TCPTP KO (Figure 2D). Together, these data demonstrate that pancreatic TCPTP deficiency mitigates cerulein-induced AP in mice.

### Pancreatic TCPTP deficiency regulates cerulein-induced STAT3 and MAPKs signaling

**Figure 3:**
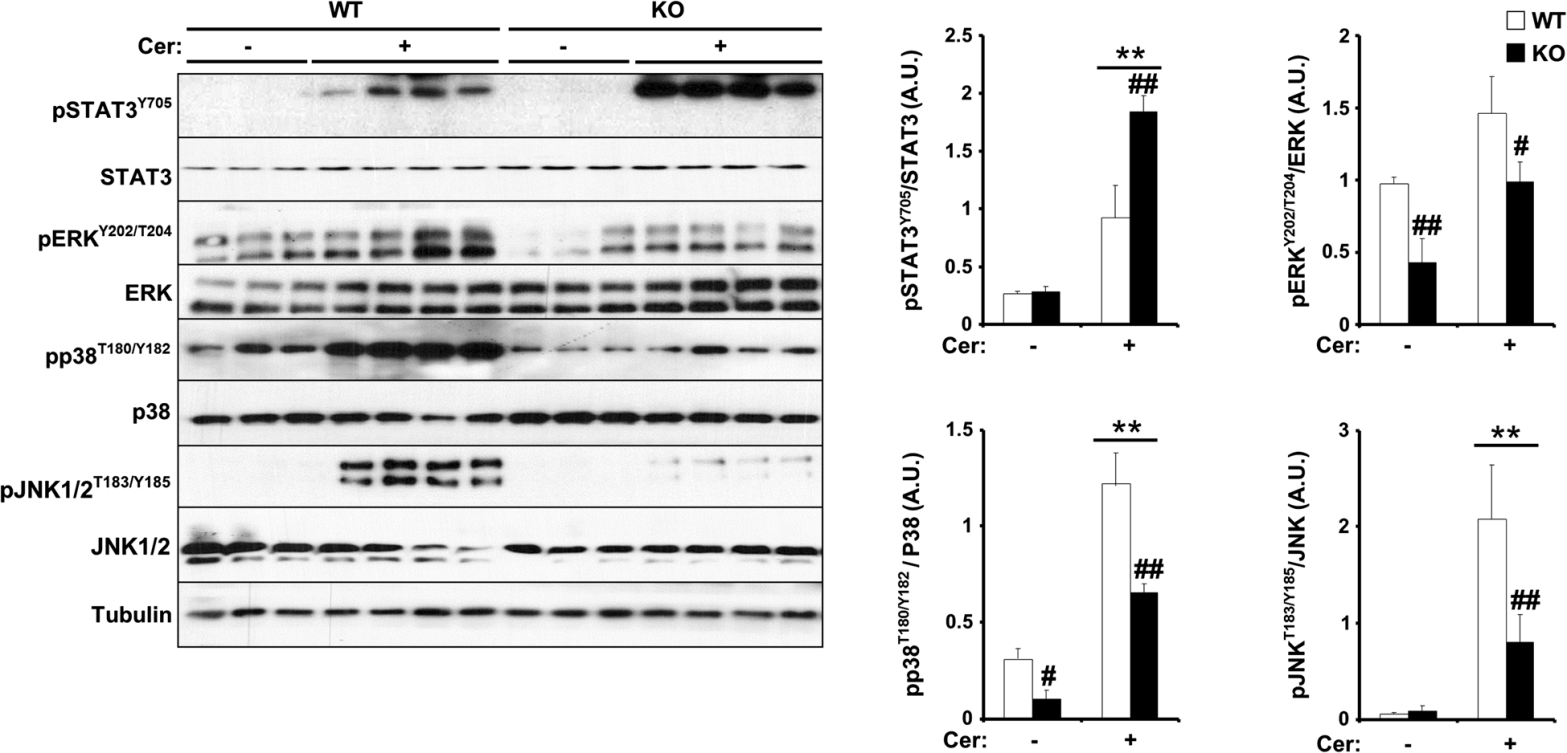
**Pancreatic TCPTP deficiency regulates cerulein-induced STAT3 and MAPKs signaling.** Total pancreas lysates from control without (n = 9) and with (n = 12) cerulein and panc-TCPTP KO without (n = 7) and with (n = 9) cerulein were immunoblotted for pSTAT3, pERK1/2, pp38, pJNK1/2 and their respective unphosphorylated proteins, and Tubulin as a loading control. Bar graphs represent normalized data for pSTAT3/STAT3, pERK/ERK, pp38/p38, and pJNK/JNK, and presented as means ± SEM. (**: P ≤ 0.01) indicates significant difference between saline- and cerulein-injected mice, and (#: P ≤ 0.05; ##: P ≤ 0.01) indicates significant difference between WT and KO mice.

To investigate the molecular basis for decreased AP in panc-TCPTP KO mice, we initially determined tyrosyl phosphorylation status of STAT3, a *bona fide* TCPTP substrate [18,26,29,30]. It is noteworthy that ablation of pancreatic STAT3 exacerbates cerulein-induced pancreatitis and demonstrates a protective effect of STAT3 against pancreatitis [45]. STAT3 is activated by phosphorylation at Tyr705 leading to dimerization and relocation to the nucleus to promote gene expression [46]. Immunoblots of total pancreatic lysates revealed significantly increased cerulein-induced STAT3 Tyr705 phosphorylation in panc-TCPTP KO mice compared with controls (Figure 3). Mitogen-activated protein kinases (MAPKs) including ERK1/2, p38 and JNK1/2 are induced rapidly and transiently during experimental AP in rodents [47]. This activation is believed to be a component of the cellular stress response in the onset of inflammation in the pancreas. Indeed, cerulein administration led to increased phosphorylation of ERK1/2, p38 and JNK in control mice that was significantly lower in panc-TCPTP KO mice (Figure 3). The decreased MAPK activation is in keeping with the reduced cerulein-induced AP and inflammation in panc-TCPTP KO mice. These findings demonstrate increased STAT3 phosphorylation and decreased MAPKs activation in pancreata of cerulein-treated panc-TCPTP KO mice.

### Pancreatic TCPTP deficiency decreases cerulein-induced NF-κB inflammation, ER stress and cell death

**Figure 4:**
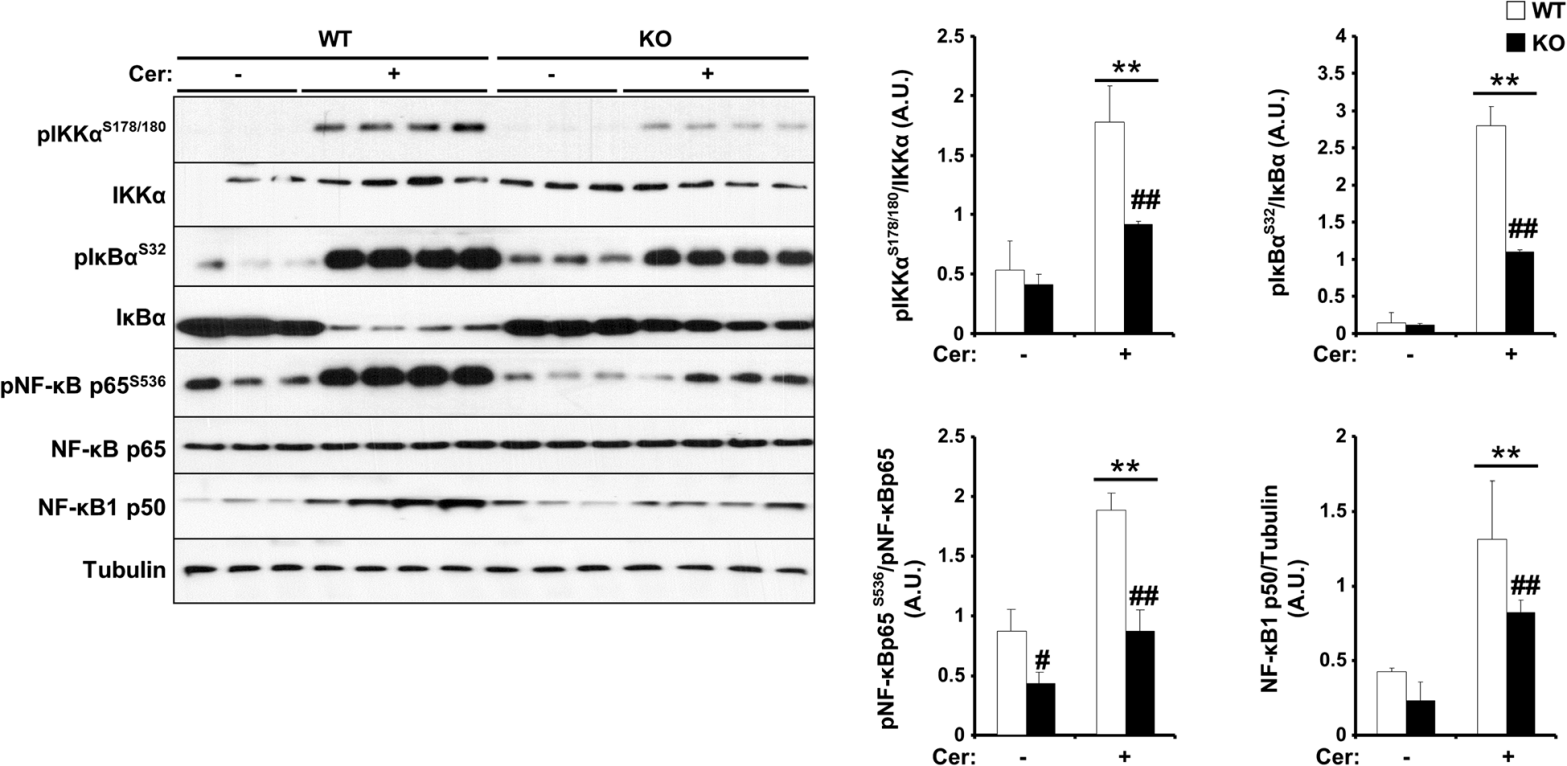
**Regulation of cerulein-induced NF-κB inflammatory response by TCPTP.** Total pancreas lysates from control without (n = 9) and with (n = 12) cerulein, and panc-TCPTP KO without (n = 7) and with (n = 9) cerulein were immunoblotted for pIKKα, pIκBα, pNF-κB and their respective unphosphorylated proteins, NF-κB1 p50, and Tubulin as a loading control. Bar graphs represent normalized data for pIKKα/IKKα, pIκBα/IκBα, pNF-κB/NF-κB and NF-κB1 p50/Tubulin as means ± SEM. (**: P ≤ 0.01) indicates significant difference between saline- and cerulein-injected mice, and (#: P ≤ 0.05; ##: P ≤ 0.01) indicates significant difference between WT and KO mice.

NF-κB is a transcription factor that regulates the inflammatory response and plays a crucial role in the pathogenesis of AP [48,49]. NF-κB is activated early in AP in leukocytes and pancreatic acinar cells [50]. Pro-inflammatory cytokines such as TNFα activate the IκB kinase complex (IKK) (composed of three subunits IKKγ, IKKα and IKKβ) to phosphorylate inhibitor of NF-κB (IκB) [51]. IκB phosphorylation triggers its ubiquitination and subsequent degradation, leading to the dissociation of NF-κB dimers (typically composed of RelA/p65 and p50) and their translocation to the nucleus for the activation of transcription [52]. Accordingly, we determined the activation status of components of NF-κB signaling pathway in control and panc-TCPTP KO mice. Cerulein-induced IKKα, IκBα and NF-κBp65 phosphorylation and NF-κBp50 expression were attenuated in panc-TCPTP KO mice compared with controls (Figure 4). These data demonstrate a decreased cerulein-induced NF-κB inflammatory response in panc-TCPTP KO mice. This is in keeping with the reduced pancreatic and circulating pro-inflammatory cytokines evident in cerulein-treated panc-TCPTP KO mice.

**Figure 5:**
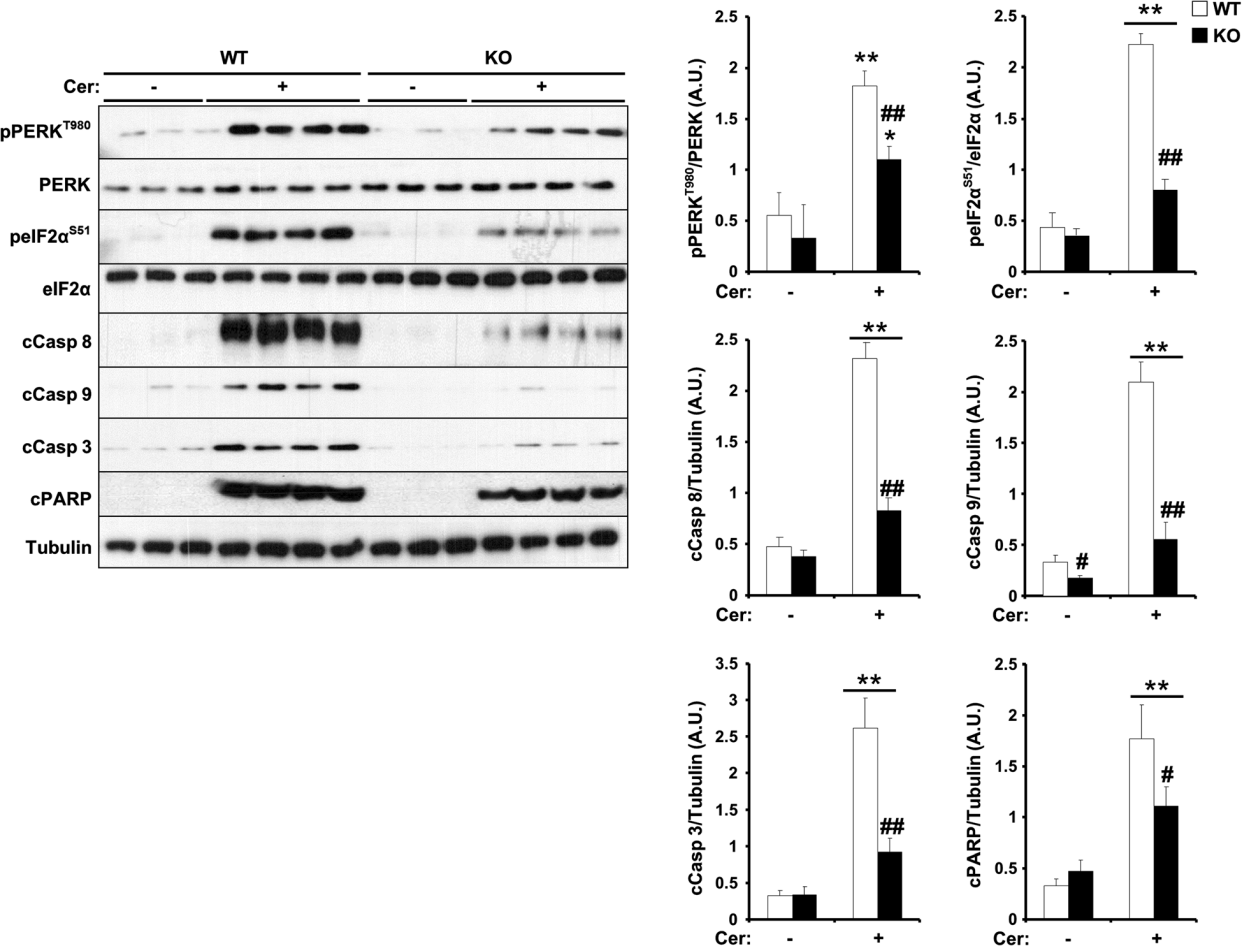
**Regulation of cerulein-induced ER stress and apoptosis by TCPTP.** Total pancreas lysates from control without (n = 9) and with (n = 12) cerulein and panc-TCPTP KO without (n = 7) and with (n = 9) cerulein were immunoblotted for pPERK and peIF2α and their respective unphosphorylated proteins, cleaved Caspases 8, 9 and 3, PARP and Tubulin as a loading control. Bar graphs represent normalized data for pPERK/PERK, peIF2α/eIF2α and Caspase 8, 9, 3 and PARP/Tubulin as means ± SEM. (*: P ≤ 0.05; **: P ≤ 0.01) indicates significant difference between saline- and cerulein-injected mice, and (#: P ≤ 0.05; ##: P ≤ 0.01) indicates significant difference between WT and KO mice.

When the folding capacity of the ER is exceeded, misfolded proteins accumulate and lead to ER stress [53]. Cells use adaptive mechanisms to mitigate ER stress known as the unfolded protein response (UPR) [54]. UPR is triggered by transmembrane sensors such as PKR-like ER-regulated kinase (PERK) that detect unfolded proteins in the ER and convey information through their cytosolic domain [55]. ER stress is implicated in the pathophysiology of pancreatitis [56]. Further, we previously demonstrated that TCPTP knockdown in the glucose-responsive MIN6 β-cells attenuated PERK-eIF2α signaling [35]. In line with these findings, pancreatic TCPTP deficiency attenuated cerulein-induced PERK Thr980 and eukaryotic translation initiation factor 2α (eIF2α) Ser51 phosphorylation compared with controls (Figure 5). The UPR is deployed by cells as a compensatory mechanism to restore homeostasis, but if it fails then apoptosis commences [57]. After exposure to apoptotic stimuli, cells activate initiator Caspases (Caspases 8 and 9) that proteolytically cleave and activate effector Caspases (Caspases 3 and 7) to dismantle dying cells [58,59]. Accordingly, we assessed cerulein-induced expression of initiator and effector Caspases in control versus panc-TCPTP KO mice. Cerulein caused pro-Caspases 8, 9 and 3 cleavage and cleavage of poly ADP ribose polymerase (PARP) (Figure 5). TCPTP deficiency decreased cleaved Caspase 8, 9 and 3 expression as well as PARP indicative of decreased apoptosis (Figure 5). Collectively, these findings demonstrate decreased inflammatory signaling, and decreased ER stress and cell death upon pancreatic TCPTP deficiency during the early phase of cerulein-induced AP.

## Discussion

The multistep development of AP involves a complex cascade of local and systemic events that occur in response to stress by the acinar cells, but the underlying cellular and molecular mechanisms are not well understood. In this study we investigated the role of TCPTP in AP using a cerulein-induced mouse model. We demonstrated increased TCPTP mRNA and protein expression during the early phase of AP. Importantly, pancreatic TCPTP deficiency in mice mitigated the effects of cerulein-induced AP. At the molecular level, panc-TCPTP KO mice exhibited enhanced cerulein-induced STAT3 tyrosyl phosphorylation, decreased NF-κB inflammatory response, and decreased ER stress and cell death. These findings uncover a novel role for pancreatic TCPTP and suggest that its pharmacological inhibition may be of value for treating AP.

Alterations in gene and protein expression during the initiation phase of AP play an important role in the development and severity of the disease [60]. In this regard, we report an increase in TCPTP expression in a cerulein-induced AP mouse model. This model was employed since secretagogue-induced pancreatitis, elicited by administration of supramaximally stimulating dose of cerulein, is the most well characterized of the pancreatitis models and has characteristics that are similar to those of human pancreatitis [8]. Using the cerulein-induced model, it was demonstrated that the expression of the SH2 domain containing phosphatases, SHP2 and SHP1 increased in AP in rats [16]. While the increase in SHP2 expression was observed in three different *in vivo* models that of SHP1 was specific to the cerulein-induced model [16]. In this study, we additionally confirmed the increased expression of TCPTP using taurocholate-treated rats thereby establishing that its expression pattern in pancreatitis is not specific to one rodent model. Similar to TCPTP, expression of the closely-related PTP1B was increased in cerulein-induced pancreatitis in mice and rats (Figure 1) [17], in contrast to the differential expression of these PTPs in the pancreata of mice after chronic high fat feeding [35]. Cerulein administration modulates pancreatic tyrosyl phosphorylation [14,15], highlighting the relevance of this signaling modality to pancreatitis and the need to further investigations on the expression and activities of PTKs and PTPs during the initiation and development of this disease. Further, SHP-1, SHP-2 and PTP1B have all been implicated in the dephosphorylation and inactivation of JAK PTKs [61-63]. Thus, it would be of considerable interest to determine whether the elevated SHP-1, SHP-2 and PTP1B act in concert with TCPTP for the coordinated inactivation of JAK/STAT3 signaling.

Using a genetic approach, we demonstrated that ablation of TCPTP in the pancreas ameliorated the course of AP as shown by the reduced serum amylase and lipase activities, decreased pancreatic *TNFα*, *IL-1β* and *IL-6* expression and decreased serum levels of TNFα and IL-6. These pro-inflammatory cytokines play a pivotal role in the development and severity of the disease [42-44,64]. TNFα exacerbates acinar cell injury and is implicated in the spread of the inflammatory cascade to other organs leading to subsequent systemic complications. In addition, IL-1β plays an important role in the development of AP and the inhibition of its production decreases the severity of the disease. Moreover, IL-6 is a major mediator of the acute-phase response and its levels correlate with the severity of the disease. Suppression of these pro-inflammatory cytokines could attenuate the severity of pancreatitis [65]. It remains unclear if the decreased expression of such pro-inflammatory cytokines in panc-TCPTP KO mice may be associated with alterations in the expression of anti-inflammatory cytokines such as IL-10. Additional studies are warranted to determine the effects of TCPTP deficiency on cytokines levels and the progression of AP.

Pancreatic TCPTP deficiency modulated cerulein-induced STAT3 phosphorylation, MAPK signaling and the NF-κB inflammatory response. STAT3, a *bona fide* TCPTP substrate [26,29], regulates the expression of genes involved in inflammatory reactions induced in response to tissue injury and infection [66]. Importantly, genetic ablation of pancreatic STAT3 exacerbates the course of cerulein-induced AP demonstrating a protective effect of STAT3 against necrotizing pancreatitis [45]. Thus, it is conceivable that the protective effects of pancreatic TCPTP deficiency in AP might be mediated, at least in part, by increased STAT3 activation. However, it is important to note that TCPTP deficiency impacted on additional signaling pathways that have been implicated previously in pancreatitis. In particular, pancreatic TCPTP deletion correlated with decreased activation of the MAPKs JNK, p38 and ERK1/2 indicative of decreased cellular stress, and is in line with previous studies implicating MAPKs in AP [67-70]. Moreover, the NF-κB inflammatory response, which plays an important role in the early stages of AP pathogenesis [48-50] was also attenuated in panc-TCPTP KO mice. The precise mechanism by which TCPTP-deficiency attenuates MAPK and NF-κB signaling remains unclear, but may be indirect and related to overall reduction in inflammation. Finally, ER stress has also been implicated in the pathophysiology of pancreatitis; the UPR attenuates alcohol-induced pancreatic damage [56], whereas PERK-deficiency impacts on the viability of the exocrine pancreas [71]. The attenuated PERK-eIF2α phosphorylation and apoptosis observed herein upon pancreatic TCPTP deficiency are in line with our previous findings implicating STAT3 in the regulation of the UPR in MIN6 cells [35] and likely contribute to the attenuated cerulein-induced pancreatic damage.

Although our studies suggest that the targeted inhibition of TCPTP in the pancreas may represent a plausible approach for combating AP, it is important to note that TCPTP is generally considered to be a negative regulator of the inflammatory response. Mice with a global deficiency in TCPTP die soon after birth from hematopoietic defects [31,72] and the development of progressive systemic inflammatory disease [32]. Moreover, T cell-specific TCPTP KO mice develop an effector/memory T cell phenotype, inflammation and autoimmunity with age [33], whereas TCPTP-deficient T cells promote autoimmunity and colitis when transferred into lymphopenic hosts [73]. These anti-inflammatory effects of TCPTP have been linked with the dephosphorylation of Src family kinases, including Lck to attenuate T cell signaling [33], and c-Src to attenuate TNF signaling [24], as well as the dephosphorylation of JAK1 and JAK3 [25] and varied STAT family members such as STAT1, STAT5 and STAT6 [25,27,28] to attenuate cytokine signaling. To our knowledge the results described in this study are the first to establish TCPTP’s capacity to promote the inflammatory response. We suggest that this occurs through the dephosphorylation of its substrate STAT3 [26,29,30], which like TCPTP, acts in a cell-type and tissue-dependent manner to elicit both pro- and anti-inflammatory actions.

In summary, the results presented herein demonstrate a novel role for TCPTP in acute pancreatitis and suggest that interventions designed to specifically inhibit TCPTP in the pancreas may be of value in treating this disease.

## Methods

### Animal studies

TCPTP-floxed (TCPTP^fl/fl^) mice on C57Bl/6J background were generated previously [33,34]. Pdx1-Cre mice on C57Bl/6J background were provided by Dr. D. Melton (Harvard University) [41]. Mice were maintained on a 12 h light-dark cycle in a temperature-controlled facility, with free access to water and food. Mice were fed standard laboratory chow (Purina lab chow, # 5001) at weaning. Genotyping for the TCPTP floxed allele and for the presence of Cre was performed by polymerase chain reaction (PCR), using DNA extracted from tails as previously described [33,34]. Acute pancreatitis was induced in 6 week old control and panc-TCPTP KO mice. Mice were fasted overnight then injected intraperiotoneally 12 consecutive times, at 1 h intervals, with cerulein (50 μg/kg body weight). DMSO was administered to the control group of mice as a vehicle control for cerulein administration. All animals were sacrificed 2 h after the last injection and blood was collected to determine serum amylase and lipase using ELISA kits (Sigma). Circulating serum cytokines levels were measured using a Multiplex kit from Meso Scale Discovery according to the manufacturer’s protocol. Pancreata were rapidly removed then portions were allocated for histology, RNA analysis and biochemistry. All mouse studies were conducted according to federal guidelines and approved by the Institutional Animal Care and Use Committee at University of California Davis.

Male Wistar rats were placed under deep anaesthesia with isoflurane before being treated with a solution of 3.5% sodium taurocholate in 0.9% sodium chloride. Acute pancreatitis was induced by a retrograde infusion of the solution before described. At 1 h, and 6 h after the induction of acute pancreatitis, rats were anaesthetized again and the pancreata were harvested and immediately snap-frozen in liquid nitrogen. Wistar rats were used in accordance with protocols approved by the Ethical Committee for Animal Experimentation and Wellbeing of the University of Valencia.

### Biochemical analyses

Pancreata were lysed using radio-immunoprecipitation assay (RIPA) buffer (10 mM Tris-HCl, pH: 7.4, 150 mM NaCl, 0.1% sodium dodecyl sulfate [SDS], 1% Triton X-100, 1% sodium deoxycholate, 5 mM EDTA, 1 mM NaF, 1 mM sodium orthovanadate and protease inhibitors). Lysates were clarified by centrifugation at 13,000 rpm for 10 min, and protein concentrations were determined using a bicinchoninic acid protein assay kit (Pierce Chemical). Proteins were resolved by SDS-PAGE and transferred to PVDF membranes. Immunoblotting of lysates was performed with antibodies for PTP1B (Abcam), TCPTP, SHP1, pPERK (Thr980), PERK, peIF2α (Ser51), pSTAT3 (Tyr705), STAT3, eIF2α, cleaved Caspases 8, 9 and 3, Tubulin (Santa Cruz), pp38 (Thr180/Tyr182), p38, pJNK (Thr183/Tyr185), JNK, p-IKKα/β (Ser178/180), IKKα/β, pIκBα (Ser32), IκBα, pNF-κBp65 (Ser536), NF-κBp65, NF-κBp50 (Cell signaling). After incubation with appropriate secondary antibodies, proteins were visualized using enhanced chemiluminescence (ECL, Amersham Biosciences). Pixel intensities of immunoreactive bands were quantified using ImageQuant 5.0 software (Molecular Dynamics).

Total RNA was extracted from pancreata using TRIzol reagent (Invitrogen). cDNA was generated using high-capacity cDNA Archive Kit (SuperScriptTM III Reverse Transcriptase, Invitrogen). TCPTP, PTP1B, SHP1, IL1-β, IL-6 and TNFα were assessed by SYBR Green quantitative real time PCR (iCycler, BioRad) using the ∆CT method with appropriate primers (Table 1) and normalized to TATA-Box binding protein (TBP).

**Table 1:** **List of primers used to quantitate *TCPTP, PTP1B, SHP1, IL1-β, IL-6 *** **and *TNFα *** **expression**

**Gene**	**Forward 5′- > 3′**	**Reverse 5′- > 3′**
IL-1β	AGCTTCAGGCAGGCAGTATC	AAGGTCCACGGGAAAGACAC
IL-6	ACAACCACGGCCTTCCCTACTT	CACGATTTCCCAGAGAACATGTG
PTP1B	AAGTTCATCATGGGCGACTC	CTGTCTTCATCCCCACAGGT
SHP1	AGCTGGGTCCCAAGGAGTAT	CTTGAGGGTAGAGGCCATGA
TBP	TTGGCTAGGTTTCTGCGGTC	GCCCTGAGCATAAGGTGGAA
TCPTP	AGAGTGGCCAAGTTTCCAGA	CACACCATGAGCCAGAAATG
TNFα	GACGTGGAACTGGCAGAAGAG	TGCCACAAGCAGGAATGAGA

### Statistical analyses

Data are expressed as means ± standard error of the mean (SEM). Single data point comparisons were performed using Tukey’s-Kramer HSD analyses using JMP program (SAS Institute). Differences were considered significant at P ≤ 0.05 and highly significant at P ≤ 0.01.

## Abbreviations

TCPTP: T Cell Protein-Tyrosine Phosphatase; AP: Acute Pancreatitis; STAT3: Signal Transducer and Activator of Transcription 3; PERK: PKR-like ER-Regulated Kinase.

## Competing interests

The authors of this manuscript declare that they have no competing interests.

## Authors’ contributions

AB: designed and performed research, analyzed data and revised the manuscript. YX: performed research and revised the manuscript. EH: performed research and revised the manuscript. NC: performed research and revised the manuscript. SB: performed research and revised the manuscript. FW: performed research and revised the manuscript. SP: performed research and revised the manuscript. SG: performed research and revised manuscript JS: contributed reagents and intellectual input and revised the manuscript TT: contributed reagents and intellectual input and revised the manuscript FH: designed research, analyzed data and wrote the manuscript. All authors read and approved the final manuscript.

## Supplementary Material

Additional file 1: Figure S1 PTP1B and SHP1 expression in panc-TCPTP KO mice. Total pancreas lysates of wild type and panc-TCPTP KO mice without (-) and with (+) cerulein administration immunoblotted for PTP1B, SHP1, TCPTP and Tubulin. Bar graph represents expression of PTP1B and SHP1 (normalized to Tubulin) and presented as means ± SEM. (*; P < 0.05, **; P < 0.01) indicates significant difference between saline- and cerulein-injected mice, and (#; P < 0.05) indicates significant difference between WT and KO mice. Click here for file
