# Supplementary material for: Pancreatic T cell protein-tyrosine phosphatase deficiency ameliorates cerulein-induced acute pancreatitis
Source: Cell Commun Signal. 2014 Mar 10;12:13. doi: 10.1186/1478-811X-12-13 (PMC4016516; doi:10.1186/1478-811X-12-13)
Supplement: Additional file 1: Figure S1 — PTP1B and SHP1 expression in panc-TCPTP KO mice. Total pancreas lysates of wild type and panc-TCPTP KO mice without (-) and with (+) cerulein administration immunoblotted for PTP1B, SHP1, TCPTP and Tubulin. Bar graph represents expression of PTP1B and SHP1 (normalized to Tubulin) and presented as means ± SEM. (*; P < 0.05, **; P < 0.01) indicates significant difference between saline- and cerulein-injected mice, and (#; P < 0.05) indicates significant difference between WT and KO mice. [file 1478-811X-12-13-S1.pdf]

Pancreatic T cell protein-tyrosine phosphatase deficiency ameliorates cerulein-induced acute pancreatitis

Ahmed Bettaieb, Yannan Xi, Ellen Hosein, Nicole Coggins, Santana Bachaalany, Florian Wiede, Salvador Perez, Stephen Griffey, Juan Sastre, Tony Tiganis and Fawaz G. Haj

Supplementary file

Figure S1: PTP1B and SHP1 expression in panc-TCPTP KO mice. Total pancreas lysates of wild type and panc-TCPTP KO mice without (-) and with (+) cerulein administration immunoblotted for PTP1B, SHP1, TCPTP and Tubulin. Bar graph represents expression of PTP1B and SHP1 (normalized to Tubulin) and presented as means ± SEM. (*; $P \leq 0.05$, **; $P \leq 0.01$) indicates significant difference between saline- and cerulein-injected mice, and (#; $P \leq 0.05$) indicates significant difference between WT and KO mice.

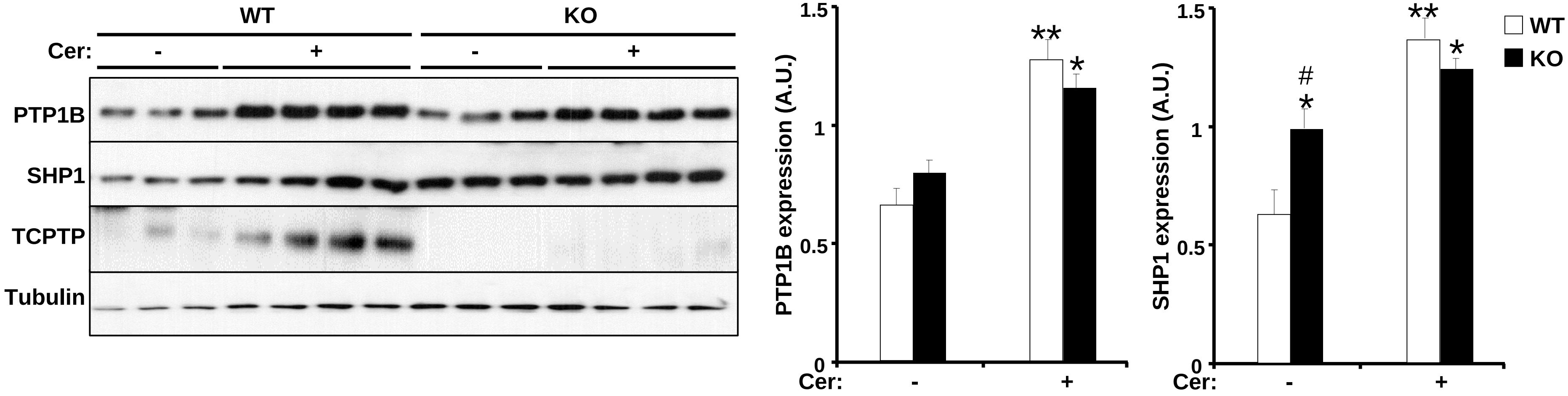

WT
KO
Cer:
-
+
-
+
PTP1B
SHP1
TCPTP
Tubulin
PTP1B expression (A.U.)
1.5
1
0.5
0
**
*
Cer:
-
+
SHP1 expression (A.U.)
1.5
1
0.5
0
#
*
**
*
Cer:
-
+
WT
KO

Figure S1
